# HNF1B-MODY (MODY-5): a rare form of diabetes with multisystemic features–two case reports

**DOI:** 10.3389/fendo.2026.1841040

**Published:** 2026-06-11

**Authors:** Tânia Carvalho, Mariana Lavrador, Joana Saraiva, Leonor Gomes

**Affiliations:** 1 Serviço de Endocrinologia, Diabetes e Metabolismo da Unidade Local de Saúde de Coimbra, Coimbra, Portugal; 2 Faculdade de Medicina da Universidade de Coimbra, Coimbra, Portugal

**Keywords:** case report, diabetes mellitus, hepatocyte nuclear factor-1beta (HNF1B), maturity-onset diabetes of the young (MODY), MODY-5

## Abstract

**Introduction:**

Maturity-Onset Diabetes of the Young (MODY) is a rare monogenic form of diabetes characterized by early onset and autosomal dominant inheritance. MODY-5, caused by HNF1B gene mutations, accounts for <5% of MODY cases and often presents with renal abnormalities and genitourinary malformations.

**Case presentation:**

We describe two patients with HNF1B-related MODY confirmed by heterozygous 17q12 microdeletions. The first case, a 29-year-old woman, presented with diabetes, hypertension, bilateral renal cysts, mild chronic kidney disease (CKD), hypomagnesemia, mild liver enzyme elevations, and a complex Müllerian anomaly requiring surgical correction. She has maintained excellent metabolic control on metformin alone, with no diabetic complications after four years of follow-up. The second case, a 40-year-old man, had a history of diabetes, neonatal left nephrectomy for cystic dysplasia, CKD stage G3bA2, persistent liver enzyme elevation, dorsal pancreatic agenesis, hypomagnesemia, hyperuricemia, mild cognitive impairment, and infertility. He initially achieved good glycemic control with a combination of insulin, metformin, liraglutide, and dapagliflozin, though control subsequently fluctuated. To date, microalbuminuria remains his only diabetes-related complication.

**Conclusions:**

These cases underscore the broad phenotypic spectrum of MODY 5 and highlight the importance of considering HNF1B mutations in young-onset diabetes associated with renal or genitourinary anomalies. Hypomagnesemia and abnormal liver function tests are additional features that may guide suspicion. Early recognition and genetic confirmation are essential for tailored management, complication surveillance, and family screening.

## 1 Introduction

Maturity-Onset Diabetes of the Young (MODY) comprises a heterogeneous group of monogenic forms of diabetes, characterized by early onset and autosomal dominant inheritance. It accounts for approximately 1-2% of all cases of diabetes, though the true prevalence is likely underestimated due to frequent misclassification as type 1 or type 2 diabetes. MODY typically presents at a young age [under 25 (or 35) years of age] and is often accompanied by a family history of diabetes, though *de novo* mutations may occur (1). Affected individuals are usually non-obese, rarely present with diabetic ketoacidosis (DKA), test negative for pancreatic islet autoantibodies, retain detectable serum C-peptide levels, and require relatively low insulin doses. To date, more than 20 genetic subtypes have been identified, each associated with mutations in different genes involved in pancreatic β-cell development, function, or glucose sensing (1).

MODY-5, also known as hepatocyte nuclear factor-1 beta (HNF1B)-MODY, is caused by HNF1B gene mutations and represents less than 5% of MODY cases (2). The HNF1B gene, located on chromosome 17q12, is expressed in the kidney, pancreatic islets, gonads, liver, lung and intestines during prenatal and postnatal development. It encodes a member of the superfamily of homeodomain-containing transcription factors which regulate gene expression and plays a critical role in the development and function of the kidneys, pancreatic β cells, liver, and genitourinary tract (3, 4)., As such, besides diabetes and pancreatic hypoplasia, MODY-5 patients usually present renal abnormalities – mainly renal cysts – and genital tract malformations. Other manifestations may include abnormal liver function test results, hypomagnesemia (due to renal magnesium wasting), hyperuricemia, early onset-gout, and neuropsychiatric disorders, such as cognitive impairment and autism spectrum disorders (5). This multisystemic presentation contrasts with most other MODY subtypes, which typically include isolated diabetes without other major extrapancreatic anomalies.

Even though this disease follows an autosomal dominant pattern, there is significant intrafamilial variability in phenotypes. Also, diabetes manifestations range widely and extrapancreatic manifestations may occur with varying degrees of severity (6). This phenotypic heterogeneity makes the diagnosis of HNF1B-associated disease challenging and requires a high index of clinical suspicion. Here, we present two cases of MODY 5 with different diabetes presentation and treatment requirements, as well as distinct systemic anomalies, highlighting the importance of considering HNF1B-related disease in patients with diabetes and the above-mentioned extrapancreatic manifestations.

## 2 Case description

### 2.1 First case

A 29-year-old woman was referred to the Endocrinology clinic for evaluation of diabetes mellitus and arterial hypertension, both diagnosed one year earlier. Her medical history included primary amenorrhea due to a complex Müllerian anomaly (two rudimentary hemiuteri and absence of vagina) treated with surgery (removal of one hemiuterus and neovagina creation), and depressive disorder. Her regular medications included metformin 700mg daily, carvedilol 6.25mg twice daily, sertraline 25mg daily and desogestrel 0, 075mg daily.

Her family history was notable for diabetes in her mother and maternal grandmother. The patient’s mother was diagnosed at age 34 years, initially treated with oral antidiabetic drugs, and required insulin therapy from age 55. The grandmother’s age at diagnosis was unknown, but she was reported to have been managed with oral medication only.

On examination, she weighed 52 kg and was 160 cm tall (BMI 20.3 kg/m²). Blood pressure (BP) was 150/100 mmHg, heart rate 87 bpm, and there was no acanthosis nigricans or other stigmata of endocrine disease.

Diabetes was detected during a routine evaluation by her primary care physician, when she reported headaches and muscle cramps. Laboratory studies revealed hyperglycemia (fasting blood glucose 134 mg/dL, 2-hour OGTT 228 mg/dL, HbA1c 6.3%), preserved C-peptide (2.5 ng/mL; reference range 1.0-7.6), and negative pancreatic autoantibodies (anti-GAD65, anti-insulin, anti-IA-2, and anti-ZNT8).

Additional testing revealed a serum creatinine of 1.22 mg/dL [estimated glomerular filtration rate (eGFR) 60 mL/min/1.73 m²], hypomagnesemia (1.5 mg/dL; reference range 1.9–2.5 mg/dL), mild hyperuricemia (7.2 mg/dL; reference range 2.6–6.0 mg/dL), and mildly abnormal liver function tests [ALT 46 U/L (<34), GGT 87 U/L (<38)]. Lipid profile was within the reference range (total cholesterol 200 mg/dL, HDL 80 mg/dL, LDL 106 mg/dL, and triglycerides 40 mg/dL).

Ambulatory blood pressure monitoring revealed persistent systolic-diastolic hypertension without nocturnal dipping (mean daytime BP 168/113 mmHg). Urinalysis, urinary sediment, protein-to-creatinine and albumin-to-creatinine ratios were all within normal limits. Ophthalmologic evaluation showed no evidence of diabetic retinopathy. An endocrine workup for secondary hypertension was negative, with normal plasma metanephrines, aldosterone-to-renin ratio, 24-hour urinary free cortisol, IGF-1, TSH, and PTH levels.

**Figure 1:**
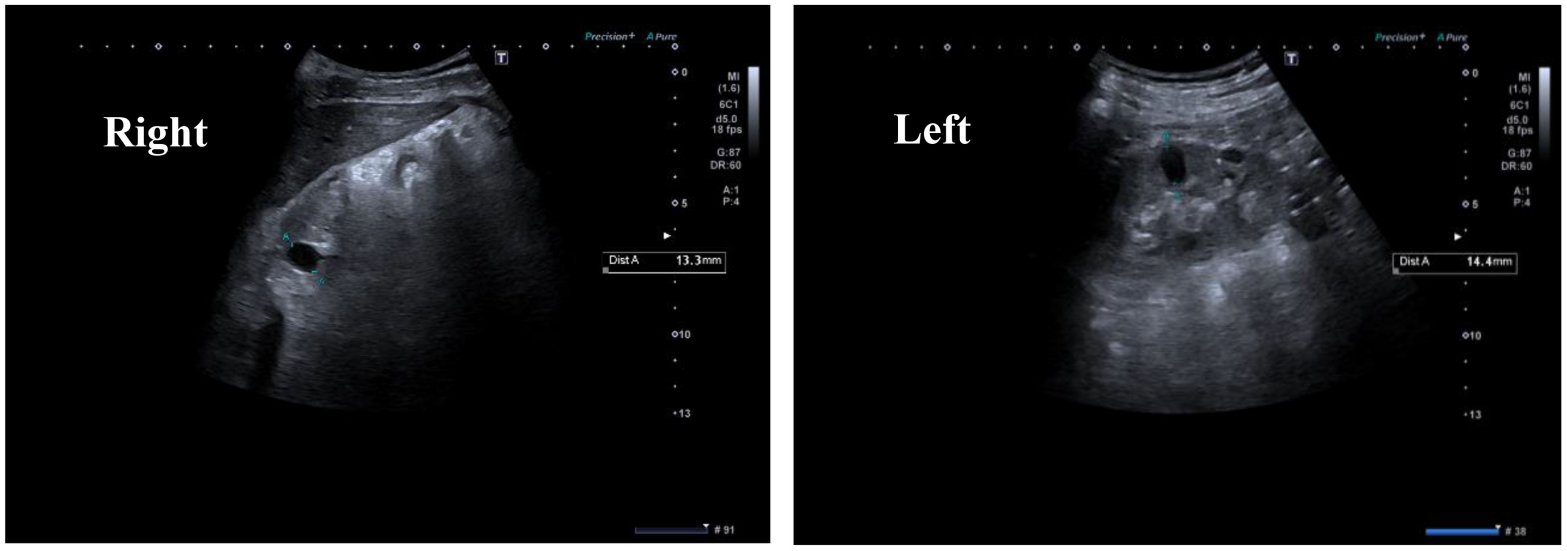
Renal ultrasound revealing bilateral renal cysts.

Renal ultrasound demonstrated bilateral simple cortical cysts, the largest measuring 14 mm on the left and 13 mm on the right, with otherwise normal kidney morphology and normal Doppler findings (**Figure 1**). Abdominal CT revealed no other anomalies, describing a normally sized pancreas with regular contours and normal parenchymal texture.

The patient was started on oral magnesium supplementation. The remaining medication was continued.

Genetic testing confirmed a heterozygous deletion of the 17q12 region, including HNF1B gene (seq[GRCh37]del(17)(q12) chr17:g.36454621-38182552del), establishing the diagnosis of MODY 5. Subsequent genetic testing of her mother revealed the same heterozygous deletion.

Over the following four years, she maintained good glycemic control (HbA1c 5.9-6.3%) and well-controlled blood pressure under the above-mentioned medication. Serum magnesium levels have fluctuated between 1.5 and 1.8 mg/dL, remaining slightly below the lower limit of the reference range. To date, no diabetic complications have been detected.

**Figure 2:**
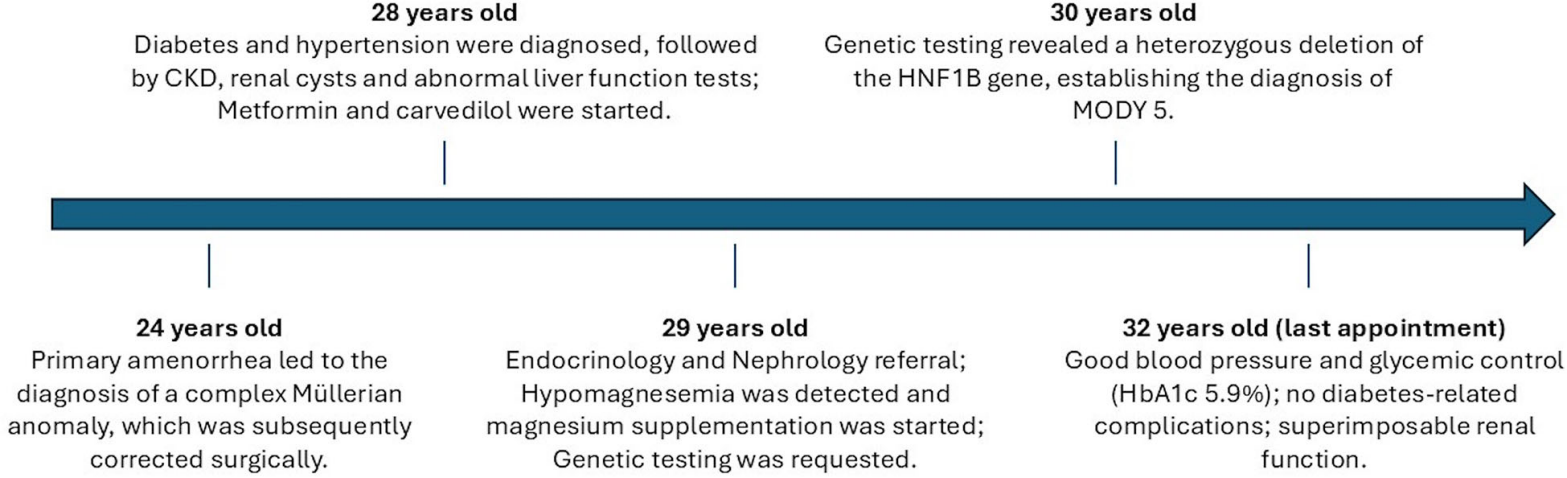
Timeline of the first patient’s clinical course. CKD, Chronic Kidney Disease.

The timeline of our first patient’s clinical course is illustrated in **Figure 2**.

### 2.2 Second case

A 40-year-old man was referred to the Endocrinology clinic for evaluation of diabetes. His past medical history was notable for left nephrectomy at 7 months of age due to polycystic kidney disease, chronic kidney disease (CKD) stage G3aA2, persistently elevated liver enzymes, mild cognitive impairment, infertility, and obstructive sleep apnea (treated with CPAP). His medications included insulin detemir 36 units in the morning and 24 units in the evening, linagliptin 5 mg daily, ramipril 5 mg daily, and atorvastatin 20 mg daily. He reported no alcohol consumption, smoking, or illicit drug use. Family history revealed type 2 diabetes in his father - diagnosed at 70 years old and treated with oral agents.

On physical examination, he was slightly overweight (BMI 25.6 kg/m²), with normal blood pressure (120/73 mmHg) and heart rate (70 bpm). No acanthosis nigricans or other stigmata of endocrine disease were noted.

He had been diagnosed with diabetes at the age of 30 years during routine laboratory testing (fasting glucose 127 mg/dL, HbA1c 6.7%). He was initially managed with oral antidiabetic drugs, achieving good glycemic control. Six years later, insulin therapy was initiated due to abrupt worsening of glycemic control (HbA1c 10.1%). Following titration of basal insulin, HbA1c improved, but frequent hypoglycemic episodes occurred, consistent with probable overbasalization.

Laboratory assessment at referral revealed HbA1c 7.7%, preserved C-peptide (1.4 ng/mL), and negative pancreatic autoantibodies (islet cell, anti-GAD65, anti-IA2). Other findings included renal impairment (serum creatinine 1.64 mg/dL, eGFR 51.2 mL/min/1.73m²), hyperuricemia (8.1 mg/dL; reference 3.5–7.2), hypomagnesemia (1.5 mg/dL; reference 1.9–2.5), markedly elevated liver enzymes [AST 102 U/L (<35), ALT 156 U/L (<45), alkaline phosphatase 472 U/L (30–120), GGT 470 U/L (<55)], and microalbuminuria (urinary albumin-to-creatinine ratio 242 mg/g; reference <30). Total bilirubin, albumin, and INR were normal. Lipid profile showed total cholesterol 125 mg/dL, HDL 40 mg/dL, LDL 71 mg/dL, and triglycerides 69 mg/dL.

Renal ultrasound demonstrated a normally sized right kidney with preserved corticomedullary differentiation, normal parenchymal thickness, and no evidence of nephrolithiasis or urinary tract obstruction.

**Figure 3:**
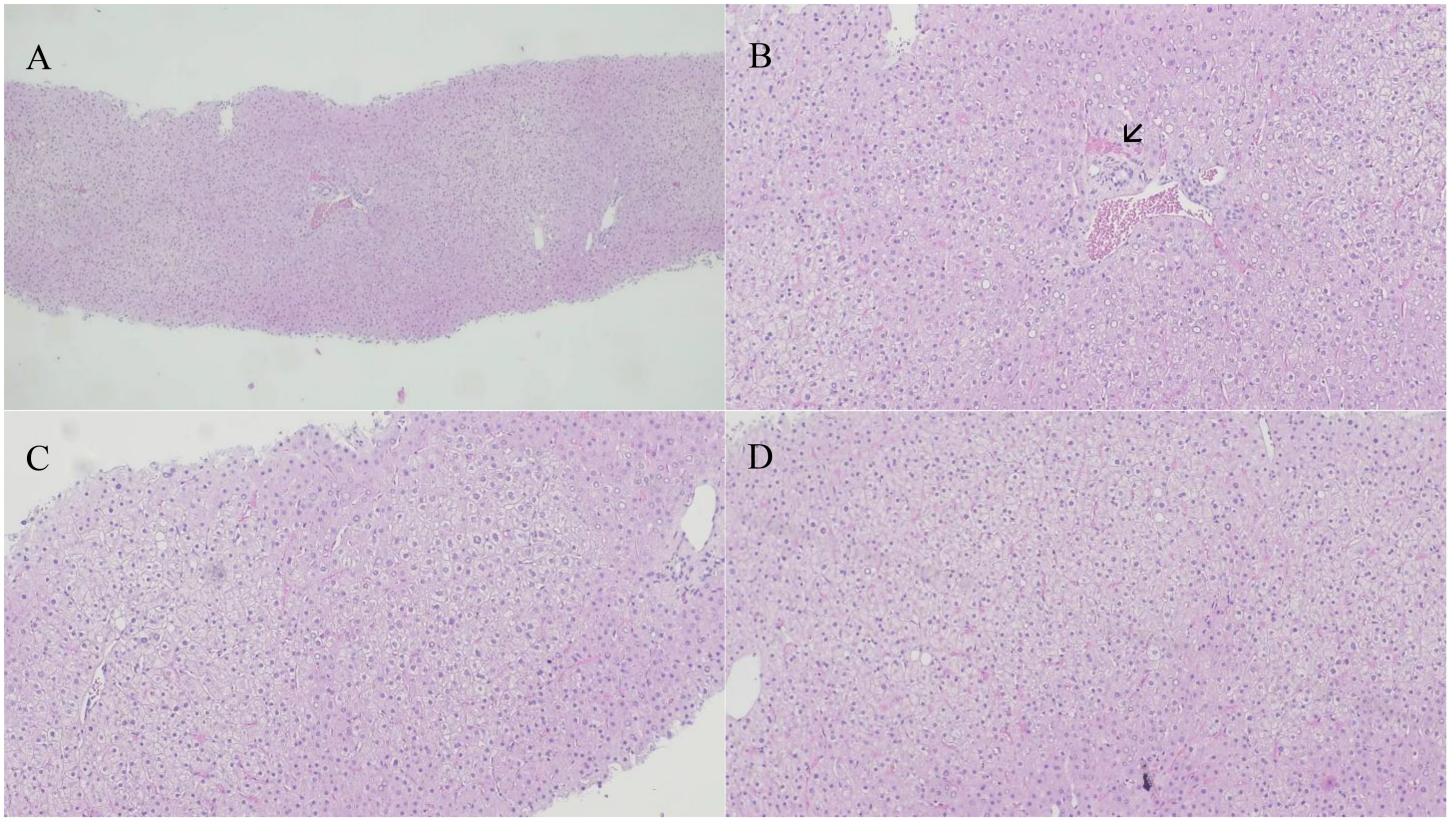
Liver biopsy. **(A)** (H&E, 40x) preserved lobular architecture with diffuse hepatocellular cytoplasmic pallor and vacuolization, without fibrosis or architectural distortion. **(B)** (H&E, 100x) prominent microvesicular steatosis with unremarkable small interlobular bile ducts (arrow). **(C, D)** mixed steatotic change with minimal macrovesicular droplets alongside predominant microvesicular steatosis, without significant inflammatory infiltrate.

Hepatological evaluation excluded viral, autoimmune, and other metabolic causes of liver disease. Abdominal CT revealed agenesis of the pancreatic body and tail, consistent with dorsal pancreatic agenesis, with no hepatic structural abnormalities. Liver elastography showed no evidence of steatosis or significant fibrosis. Histopathological analysis of a liver biopsy demonstrated preserved architecture with seven portal tracts showing full preservation of their components, diffuse predominantly microvesicular steatosis and glycogenated nuclei, without features of steatohepatitis or fibrosis (**Figure 3**).

Basal insulin doses were reduced, and therapy was optimized with metformin 500 mg twice daily, liraglutide 1.8 mg daily, dapagliflozin 10 mg daily, and prandial insulin as-needed, improving glycemic control (HbA1c 6.8%) and reducing hypoglycemia.

Given the syndromic features, a genetic study was performed. Array comparative genomic hybridization (CGH) identified a heterozygous deletion on the long arm of chromosome 17 (17q12): 46, XY.arr[GRCh37]17q12(34817422_36248918)x1, classified as pathogenic, thus confirming the diagnosis of HNF1B-related MODY.

During follow-up, glycemic control was variable due to intermittent therapeutic non-adherence, with HbA1c levels peaking at 13.2% during periods of noncompliance. At his most recent evaluation, at age 46, HbA1c was 7.3%, with continuous glucose monitoring (CGM) metrics showing time in range 40%, time above range 51%, time below range 7%, and coefficient of variation 31%. His eGFR had declined to 44 mL/min/1.73m², but albuminuria had improved (58 mg/g). No macrovascular disease, diabetic retinopathy, or neuropathy had been documented to date.

The patient continues multidisciplinary follow-up in Endocrinology, Hepatology, and Nephrology.

**Figure 4:**
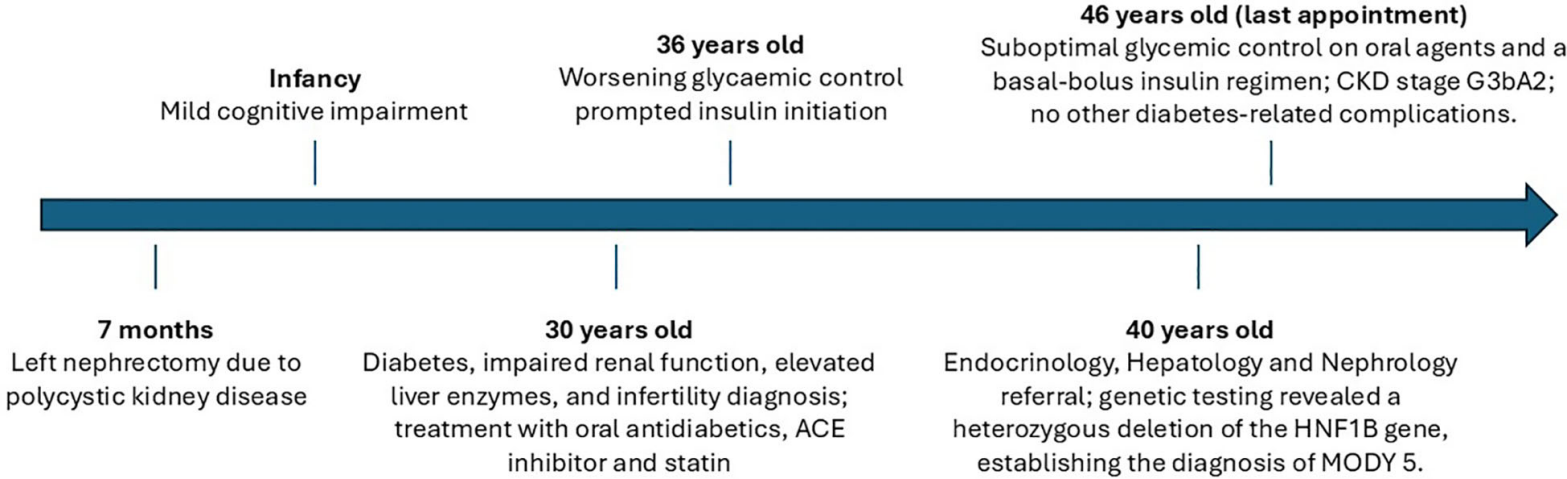
Timeline of the second patient’s clinical course. ACE inhibitor, Angiotensin-Converting Enzyme inhibitor; CKD, Chronic Kidney Disease.

The timeline of this patient’s clinical course is illustrated in **Figure 4**.

## 3 Discussion

We report two cases of MODY-5 caused by heterozygous 17q12 microdeletions, highlighting the multisystemic nature of HNF1B-associated disease and its marked phenotypic variability, even among individuals with the same genetic defect. We provide detailed characterization of metabolic, renal, and hepatic involvement, including histopathological liver findings, a poorly described feature in the literature. The contrasting clinical trajectories further underscore the heterogeneity of disease expression and contribute to the limited evidence on genotype-phenotype correlations and disease progression.

### 3.1 Diabetes phenotype in HNF1B-MODY

Diabetes occurs in about 80% of patients with HNF1B mutations and is often associated with pancreatic hypoplasia or atrophy, reported in up to 60% of cases. The pathophysiology involves primary β-cell dysfunction, although insulin resistance has also been described (7, 8). The mean age at diagnosis is around 25 years, consistent with our patients, diagnosed at 29 and 30 years, respectively. Patients tend to be leaner, as was the case of our first patient (2). Clinical presentation ranges from asymptomatic hyperglycemia, as in our patients, to classical osmotic symptoms (polyuria, polydipsia, weight loss); ketoacidosis is rare. The degree of hyperglycemia and therapeutic needs vary widely. One review showed HbA1c values at diagnosis ranging from <7% in one-third of patients to >13% in another third (7). Residual insulin secretion is usually preserved: fasting C-peptide >0.20 nmol/L or stimulated C-peptide >0.50 nmol/L is observed in 80% of patients with HNF1B mutations, in line with our cases (2.5 and 1.4 ng/mL) (6). Nevertheless, the severity of diabetes differed substantially between our two patients. The first maintained excellent glycemic control with metformin alone, whereas the second required basal-bolus insulin plus multiple oral and injectable agents, yet continued to experience suboptimal control. This contrast may be explained by differences in age at presentation, residual β-cell function, and pancreatic morphology - our female patient had a normal pancreas on CT, whereas the male patient had dorsal pancreatic agenesis. Although around 50% of MODY 5 patients initially respond to oral agents, insulin dependency develops in up to 80% within 10 years. This is due to the progressive β-cell failure and declining renal function (6). In a study by Dubois-Laforgue et al., microvascular complications were detected in 40% of patients after a 15 year-observation period (7). Presently, neither of our patients shows evidence of macro or microvascular complications with exception of microalbuminuria in the second case, likely reflecting longer disease duration, worse glycemic control, and more advanced CKD.

### 3.2 Renal manifestations

Renal anomalies are the most consistent feature of MODY 5, affecting ~90% of patients with HNF1B mutations. Multicystic disease is the most frequent, but other abnormalities such as hyperechogenic kidneys, poor corticomedullary differentiation, hydronephrosis, and ureteral dilatation have been described. Renal function ranges from normal (in 59%) to CKD (in 41%) (5, 7). Both of our patients had cystic kidney disease and CKD, albeit with differing severity: the first had simple bilateral cysts and CKD G2A1, whereas the second underwent neonatal left nephrectomy for cystic dysplasia and currently has CKD G3bA2. Close monitoring is recommended, as this disease leads to end-stage renal disease (ESRD) in a considerable number of patients, including hemodialysis and kidney transplantation (7).

Hypertension was present in our first case but not the second. After extensive evaluation, no other secondary causes were identified. Although renal function was only mildly impaired, the most plausible explanation was renal-mediated hypertension.

### 3.3 Electrolyte and metabolic abnormalities

Hypomagnesemia, resulting from renal magnesium wasting, is observed in up to 75% of patients with *HNF1B* mutations and may occasionally be one of the predominant features. It may be asymptomatic or cause neuromuscular, neuropsychiatric, gastrointestinal symptoms, or cardiac arrhythmias, and has been linked with insulin resistance, poorer glycemic control, and increased cardiovascular risk in individuals with diabetes (9). Although supplementation has not yet proved to improve cardiovascular outcomes, surveillance and treatment should be pursued. In both of our cases, hypomagnesemia persisted below the reference range despite supplementation, consistent with previous reports describing the difficulty in fully correcting this abnormality (7, 10).

Hyperuricemia occurs in up to 65% of individuals with HNF1B mutations and may manifest early in life, with some patients developing gout. Therapy with allopurinol has been proposed to help control serum uric acid levels and potentially delay renal disease progression (2). Hyperuricemia was present in both our patients, although neither developed gout.

### 3.4 Hepatic involvement

Abnormal liver function tests are reported in nearly 70% of patients and may, in some cases, manifest as cholestatic liver disease. Liver imaging abnormalities are less common, occurring in approximately 30% of cases. Although rare, severe cholestasis progressing to end-stage liver disease and requiring liver transplantation has been described. In our series, the female patient had only mild ALT and GGT elevations, with normal hepatic imaging. In contrast, the male patient exhibited more pronounced hepatic involvement, with persistent elevations of transaminases, alkaline phosphatase, and GGT. Hepatic imaging was unremarkable, but liver biopsy demonstrated diffuse predominantly microvesicular steatosis and glycogenated nuclei, without features of steatohepatitis or fibrosis, and preserved portal tracts. Hepatic involvement in HNF1B-related disease remains poorly characterized, as most data rely on biochemical abnormalities, with limited histological correlation. Previous reports have often noted a reduced numbers of intrahepatic bile ducts, and less frequently fibrosis, steatosis, and focal nodular hyperplasia, underscoring the important role of *HNF1B* in biliary and hepatic development (7, 11).

### 3.5 Genitourinary and reproductive features

Genital tract malformations are reported in up to 50% of patients with HNF1B mutations (2). Female patients may present with bicornuate uteri, rudimentary uterus or vaginal aplasia, while male patients may exhibit cryptorchidism, varicocele, hypospadias, prostatic hypoplasia, agenesis of the vas deferens, and asthenospermia (11, 12). Our female patient had a complex Müllerian anomaly with rudimentary hemiuteri and complete vaginal agenesis, requiring surgical correction. The male patient reported infertility, a feature also associated with HNF1B mutations, although no structural anomaly was identified.

### 3.6 Neurological features

Neurodevelopmental involvement has been described in some patients with MODY-5, including global developmental delay, intellectual disability, learning disorders, attention deficit/hyperactivity disorder, autism spectrum disorder, schizophrenia and bipolar disorder. These manifestations appear to be more frequent in individuals with 17q12 deletions than in those with HNF1B mutations, suggesting that loss of additional genes within the deleted region may aggravate the neuropsychiatric phenotype. Notably, marked phenotypic heterogeneity has been reported even among family members with identical deletions (13). In our series, the second patient had mild cognitive impairment that affected treatment adherence, whereas the first exhibited no neurological abnormalities, further illustrating this variability.

A summary table comparing the phenotypic features of our patients with those reported in the literature is provided (**Table 1**).

**Table 1:** Comparison of phenotypic features in HNF1B-MODY.

Feature	Case 1	Case 2	Reported frequency in literature
Diabetes	Yes	Yes	~80%
Age at diagnosis	28	30	~25 years (mean)
Renal abnormalities	Bilateral cysts + CKD	Nephrectomy (polycystic disease) + CKD	~90%
CKD	Mild	Moderate	~40%
Hypomagnesemia	Yes	Yes	~75%
Hyperuricemia	Yes	Yes	~65%
Liver involvement	Mild enzyme ↑	Severe enzyme ↑ + Steatosis	~70%
Pancreatic morphological abnormalities	None	Dorsal agenesis	~60%
Genital anomalies	Müllerian anomaly	Infertility	~50%
Neurocognitive features	No	Mild impairment	Variable

### 3.7 Genetic considerations

Genetic testing is essential to establish the diagnosis of MODY-5. Approximately half of cases are caused by large heterozygous 17q12 microdeletions, spanning 1.5 Mb on average and encompassing 15 genes, including HNF1B. In the remaining half, punctual mutations in HNF1B gene, such as missense, nonsense, small deletions or insertions, frameshift and splicing mutations, are responsible for the disease (7). Emerging evidence suggests that these two genetic mechanisms may be associated with partially distinct phenotypic patterns. Whole-gene deletions often encompass multiple genes and have been linked to neurodevelopmental disorders, a more severe phenotype of diabetes, requiring insulin more frequently, as well as relatively preserved renal function and a lower likelihood of kidney transplantation (7, 13). Despite these tendencies, no robust genotype-phenotype correlation has been established, as both large deletions and point mutations can result in multisystemic disease with highly variable expression. Importantly, deletions may not be detectable by conventional sequencing methods such as whole-exome sequencing or targeted gene panels, requiring copy-number-sensitive methods such as multiplex ligation-dependent probe amplification (MLPA) or array-CGH for diagnosis. Consequently, a negative result on standard sequencing does not exclude HNF1B-associated disease, and additional copy-number analysis should be pursued when clinical suspicion is high. (14, 15).

### 3.8 Clinical presentation, progression, and management implications

The initial clinical presentation of HNF1B-related disease is highly variable and may precede the diagnosis of diabetes by several years (4). In our first case, the earliest manifestation was primary amenorrhea due to a complex Müllerian anomaly, whereas in the second case it was cystic renal disease requiring nephrectomy during the first year of life. In previously reported series, other initial presentations include chronic kidney disease, severe hypomagnesemia, or elevated liver enzymes (10, 13).

Disease progression is heterogeneous and not fully predictable. Glycemic control may remain stable for years or deteriorate more rapidly due to progressive β-cell dysfunction, although most patients will eventually require insulin therapy (7, 11). The decline in renal function is similarly variable, does not always correlate with the severity of structural abnormalities on imaging, and may progress to end-stage kidney disease in some patients (2). The trajectory of other systemic features, including hepatic and neurocognitive involvement, remains less well defined.

Given its multisystemic nature, management requires a multidisciplinary approach. In addition to individualized glycemic treatment and regular renal monitoring, clinicians should actively screen for associated comorbidities. When identified, patients should be referred to the appropriate specialties, including nephrology, endocrinology, hepatology, reproductive medicine, gynecology or urology, medical genetics, and, when indicated, psychiatry or psychology (13). Early recognition and coordinated care are essential to optimize outcomes and enable appropriate family counseling.

## 4 Conclusions

MODY-5 is a multisystemic disorder with variable clinical expression. These cases highlight the importance of considering MODY-5 in young-onset diabetes associated with renal abnormalities and/or genital tract malformations. Other features that may point to this diagnosis include hypomagnesemia, abnormal liver tests and neuropsychiatric disorders. Because conventional sequencing may fail to detect HNF1B deletions, copy-number analysis should be pursued when clinical suspicion is high. Early recognition and genetic confirmation are crucial to guide individualized management, enable surveillance for extrapancreatic comorbidities, and allow family counseling and screening.

## Data Availability

The original contributions presented in the study are included in the article/supplementary material. Further inquiries can be directed to the corresponding author.
